# Coenzyme Autocatalytic Network on the Surface of Oil Microspheres as a Model for the Origin of Life

**DOI:** 10.3390/ijms10041838

**Published:** 2009-04-22

**Authors:** Alexei A. Sharov

**Affiliations:** Genetics Laboratory, National Institute on Aging, NIA/NIH / 251 Bayview Boulevard, Baltimore, MD 21224, USA; E-Mail: sharoval@mail.nih.gov; Tel. +1-410-558-8556; Fax: +1-410-558-8331

**Keywords:** Origin of life, coenzyme world, RNA world, hydrocarbon, coding relation

## Abstract

Coenzymes are often considered as remnants of primordial metabolism, but not as hereditary molecules. I suggest that coenzyme-like molecules (CLMs) performed hereditary functions before the emergence of nucleic acids. Autocatalytic CLMs modified (encoded) surface properties of hydrocarbon microspheres, to which they were anchored, and these changes enhanced autocatalysis and propagation of CLMs. Heredity started from a single kind of self-reproducing CLM, and then evolved into more complex coenzyme autocatalytic networks containing multiple kinds of CLMs. Polymerization of CLMs on the surface of microspheres and development of template-based synthesis is a potential evolutionary path towards the emergence of nucleic acids.

## 1. Introduction

Evidence that molecular components of a living cell can be synthesized abiotically is no longer sufficient for a scenario of the origin of life because combinations of these components can never make a functioning and evolving living organism. Thus, contemporary efforts in the area of life’s origin are focused on functional circuits, in particular, on metabolic networks (“metabolism first” hypotheses) and self-replicating nucleic acids (e.g., RNA) or their predecessors (“replication first” hypotheses) [1,2]. “Replication first” hypotheses can explain adaptive evolution of primordial systems via random mutations and natural selection, however they require unrealistic conditions that monomers (e.g., nucleotides) are readily available as resources for template-based synthesis. Nucleotides are complex molecules and the probability of their spontaneous synthesis is not higher than the probability of spontaneous assembly of a glass bottle or a hammer. Nucleotides were optimized for reliable storage and replication of genetic information, thus it is reasonable to expect that they emerged as a product of a long adaptive evolution [3].

“Metabolism first” hypotheses imply that autocatalytic networks can be assembled from mixtures of interacting organic molecules not capable of template-based replication. For example, it was suggested that short peptides may get organized into an autocatalytic network which can persist indefinitely if there is an abundant supply of various free aminoacids in the media [4]. One of the problems with this hypothesis is that aminoacids are not likely to be synthesized abiotically in sufficient quantities to support autocatalysis. To avoid this problem, it was suggested that first autocatalytic networks consumed only simple abundant resources. For example, the reverse citric acid cycle can in theory reproduce all its components from fixation of carbon dioxide [5]. This scenario has been criticized from the biochemical point of view [6]. However, even if the chemistry of the cycle worked properly, it is not clear how this network could evolve via mutations and selection because it was not enclosed in a proto-organism.

The discussion on whether metabolism or replication emerged first is often viewed as misleading and similar to the “chicken and the egg” puzzle [3]. Thus, there were several attempts to make a synthesis of these approaches. Konnyu *et al*. proposed that life started from self-replicating RNA coupled with encoded metabolic network, which is needed to produce all necessary monomers [7]. However, an organism that combined both nucleic acids and metabolism is too complex to emerge spontaneously from a random mixture of molecules [8]. Copley *et al*. proposed that “mutual catalysis in proto-metabolic reaction networks led, perhaps inexorably, to the emergence of RNA as a dominant macromolecule that supplied both catalysis and genetic information” [3]. This scenario is more realistic because it starts from simple autocatalytic circuits, which could have existed in hydrothermal circulations. Copley *et al*. noted that at early stages of evolution, self-replication was implemented at the level of the entire metabolic network, rather than for individual molecules [3]. However, they have not defined the notion of replication or coding and did not show which kind of molecules supported the coding function before the emergence of nucleic acids. Dyson suggested a “double-origin hypothesis” which assumed that metabolism and replication originated separately in two kinds of proto-organisms, and then replication took over metabolism in a similar way as parasites take over host organisms [9]. Wächtershäuser proposed that autocatalytic networks of organic molecules are more likely to emerge on positively-charged mineral surfaces than in water solution [10]. This “surface metabolism” is partially separated from its 3-dimensional environment, and, according to Wächtershäuser, it can evolve via inherited variation of autocatalytic coenzymes and selection.

In this paper I follow the general approach of Copley *et al*. [3] and Wächtershäuser [10] by assuming that metabolism and coding emerged simultaneously and started from simple non-polymeric molecules capable of autocatalysis. In this context, I elaborate the notion of coding, which is the essential property of life. Coding is defined as a triadic relation between organisms, their coding elements (CE), and encoded functions. In contrast to single-level metabolic networks, the coding relation unites two hierarchical levels: the organism and its CEs. Evolution of proto-organisms was based on acquisition or modification of functions encoded by novel or modified CEs. Because these functions affected the survival and reproduction of proto-organisms, the evolution was guided by selection towards increased population growth rates and more efficient use of resources. Polymeric CEs and subsequent template-based copying mechanisms emerged later and improved gradually under the control of selection.

Although we do not know the chemical structure of simple CEs that preceded the origin of nucleic acids, it is reasonable to assume that they were similar to contemporary coenzymes [10]. Coenzymes are often considered as remnants of primordial metabolism [11], however they are not usually considered as hereditary molecules. The proposed model of the origin of life assumes that coenzyme-like molecules were anchored to the surface of oil microspheres where they performed both autocatalytic and heterocatalytic activities. Autocatalysis ensured heredity (i.e., self-production of CEs), whereas heterocatalysis supported the encoded function (e.g., modification of surface properties of the microsphere). Such systems, which I call “coenzyme world”, are simple enough to emerge spontaneously, they do not require any advanced organic molecules as resources, and can be viewed as predecessors of more complex organisms with polymerized CEs capable of template copying.

## 2. Results: “Coenzyme World” Model of the Origin of Life

### 2.1. Definition of Coding Relation

Here I use the term “coding” in a broad sense, not just as a correspondence between triplets of nucleotides in mRNA and aminoacids in the protein, but as the ability of living systems to store recipes on how to perform various functions from cellular metabolism to complex animal behavior [12,13]. For example, epigenetic stability and neural memory represent the short-term storage of encoded functional information, whereas heredity represents the long-term storage of information which extends over many generations [14]. Stability of encoded functional information is supported by the organism and is implemented as a conditional autocatalysis of CEs. The definition of autocatalysis as a reaction, whose rate is positively affected by one of the products, is not sufficient to characterize CEs. CEs are irreplaceable, which means that they cannot be recovered if all copies are destroyed. Thus, CEs cannot be captured easily from the environment and require strong autocatalysis, whose rate is zero in the absence of products. CEs are products of the autocatalysis and should not be confused with resources (e.g., precursors), which are reagents. Resources are simpler and more abundant molecules than CEs, whereas CEs are either rare molecules or do not exist in the inanimate world. Genes are examples of polymeric CEs, which are duplicated via template-based copying process. In contrast, primitive CEs are relatively simple molecules, which catalyze their own synthesis, and therefore produce copies without template matching. Both mechanisms of copying are strict autocatalytic reactions because an existing CE is required for generating copies. Association of copying with autocatalysis may seem confusing because humans can copy almost anything. However, in living organisms, very few kinds of molecules can be copied even with external help; and at the time of life’s origin, this external help was minimal.

CEs are produced via either autocatalytic reaction or autocatalytic cycle. In the latter case, the reaction rate is positively affected by products generated after several downstream reactions rather than by the immediate products. Molecules at all phases of the autocatalytic cycle are copies of the same CE because they produce each other following reactions of the cycle. Both types of autocatalysis can be formally represented by a Petri net with specific properties [15]. It appears that each self-reproducing system with these properties has a unique minimum core which is a cycle, where each component can produce any other component from that core [15]. Thus, each kind of CEs can be viewed as a minimum self-reproducing system.

Theoretically, all self-reproducing systems can support their existence. However, the viability (i.e., persistence) of such systems depends on additional external and internal factors, including abundance and accessibility of resources, degradation and diffusion rates, and time delays. These factors are inter-dependent. For example, higher rates of degradation or diffusion require abundant resources, which can support an increased rate of catalytic synthesis. Temporal changes in resource abundance may also affect the viability of the system.

**Figure 1.**
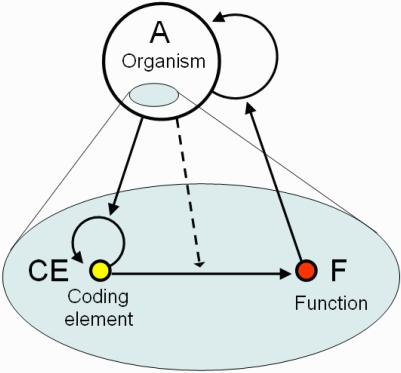
Components of the coding relation. Coding element, CE, encodes function, F, which enhances survival and reproduction of organism, A; autocatalysis of CE is mediated by A.

I define coding as a triadic relation between an organism A, its CE (which may be present in multiple copies), and function F (Figure 1), where (1) CE is a viable and irreplaceable self-reproducing system within organism A; (2) CE induces or modifies some function F (i.e., behavior/development) of the organism A; (3) survival and/or reproduction of A is supported by function F. Both CE and F are parts of A as shown in the figure, thus the coding relation unites two levels of structural-functional hierarchy. We can say that CE encodes F for organism A. Evolutionary advanced organisms can utilize coding relations at multiple time scales. For example, an epigenetic state of a specific DNA region represents a short-term CE whereas genes are long-term CEs. The coding relation has a circular structure with a positive feedback because CE encodes the function of the organism, which in turn supports self-reproduction of the organism, and the organism supports self-reproduction of the CE (Figure 1). Thus, it can be associated with the concept of semantic closure which in brief means a self-supporting interpretation of the code [16,17].

The relation between CE and F is more than just a cause-effect relation because it does not exist without organism A. It can be viewed as a semiotic relation which emerged within evolving organisms to support (i.e., “memorize”) their specific function. This relation is contingent or arbitrarily defined because the same function can be encoded in many different ways, but only one kind of CE (usually the one that appears first) is preserved and then carried through branching phylogenetic lineages. Later, the same CE can be reused for different novel functions. Studies of biological evolution have long been limited to one or two components of the code relation. Paleontology and taxonomy are focused on the organism component; genetics studies the relation between CEs (genes) and organism morphology; and evolutionary biology links functional characteristics (e.g., embryonic development and behavior) with organism’s morphology. However, all components of the code relation are equally important, and each may take a leading role in a specific episode of evolution [14]. For example, mutations of the DNA sequence is not the only driving force of evolution. Changes may start from a modified interpretation of the same DNA sequence in a changed environment or from alternative functions of a body part (e.g., Baldwin effect) [18]. However, all changes have to be encoded in order to persist through generations.

If some function is needed only at a specific stage of organism development or in a specific environment, then an organism can interpret the code for this function on demand when these conditions are met, and shut down interpretation at other times. Interpretation on demand can be illustrated by gene regulation at multiple steps including transcription, mRNA stability, translation, protein modification and localization. Interestingly, the ability to regulate the interpretation of a CE is encoded by other CEs. For example, transcription of hormone-responsive genes is regulated by hormone receptors, which are encoded by other genes.

The proposed definition of coding in living organisms challenges the idea of von Neumann that the genome is a description of an organism [19]. A code is descriptive if its meaning can be recovered (i.e., computed) from the meaning of its parts (e.g., characters, words) following known syntax rules. However, the code is not fully descriptive because it cannot be partitioned indefinitely and the meaning of parts at the lowest level is not described. This is related to the idea of Kampis that life is not computable [20]. CEs do not fully describe or compute organisms or their parts but encode methods for their production. Similarly, a recipe book helps to bake the bread without describing the final product. A recipe is based on non-descriptive indexes to components (e.g., take a cup of flour and a cup of water...). Similarly, CEs in living organisms may provide non-descriptive indexes by selective binding to specific molecules, or by producing other cellular components capable of selective binding. The advantage of a non-descriptive code is that it does not need to carry all the information, and therefore, it can be much smaller than the full description of an organism. This may seem as a quantitative difference, but it appears essential for the origin of life. If the code of first living organisms were descriptive, it would be as complex as the organism itself and would never emerge spontaneously. However, non-descriptive coding can start spontaneously from a single CE.

### 2.2. Emergence of Primordial Code-Driven Functional Systems

The first code-driven functional systems were in many respects similar to contemporary organisms: they used resources, reproduced, and encoded chemical functions that had a beneficial effect on their persistence and reproduction. Only the number of encoded functions was extremely small and most likely life started from a single function and a single kind of CEs. Emergence of the coding relation can be viewed as a start of the long process of life’s origin.

Although the chemistry of first CEs is unknown, it is reasonable to hypothesize that they were similar to contemporary co-enzymes. They could not be freely available in the environment as resources because CEs are irreplaceable by definition. Thus, they had a more complex chemistry than saturated hydrocarbons which are most abundant organic molecules and can be synthesized abiogenically [21,22]. Initial CEs had to be active catalysts because they needed to support (1) auto-catalysis, i.e. assembly of their copies from precursors, and (2) encoded function within a larger host system. Passive CEs could not appear first because there was no interpretation system yet. Because primordial CEs should be treated as evolutionary predecessors of contemporary nucleic acids, we expect that some of them were similar to nucleic acids. Among simple organic molecules in the cell, coenzymes (e.g., NADH, ATP, CoA) are most similar to nucleic acids; thus, following Wächtershäuser [10] and Danchin [23], we can hypothesize that first CEs were coenzyme-like molecules (CLMs) which were common ancestors of both nucleic acids and contemporary coenzymes. By a CLM, I mean a small non-polymeric organic molecule with catalytic activity.

Let us now reconstruct possible components of the first code relation. The role of organisms can be played by oil microspheres in a water environment. By oil I mean liquid hydrocarbons which can be present on early terrestrial planets [24] and can be synthesized abiogenically in hydrothermal vents [21, 22]. Hydrocarbons aggregate and form microspheres in water, making discrete resources that can easily support long-persisting catalytic networks. Oil microspheres can be broken mechanically into smaller parts or merged with other microspheres. In this way, catalytic networks on the surface of oil microspheres can propagate and capture new resources. The last but not the least reason for selecting oil microspheres as candidates for proto-organisms is that hydrocarbons provide a link to the future origin of bilayer lipid membranes, as discussed below. In comparison, the “lipid world” model of the origin of life [25] assumed that first proto-organisms were lipid micelles or bilayer vesicles. However, this assumption seems unrealistic because lipid molecules cannot be synthesized abiotically in sufficient quantities. Even simple amphiphilic molecules (e.g., fatty acids) were rare molecules compared to hydrocarbons before the origin of life. Thus, they were diluted by hydrocarbons and unable to form micelles or bilayer vesicles.

The function of first CEs anchored to the surface of microspheres could have been catalytic oxidation of terminal carbon atom in hydrocarbon molecules. Because oxidized terminals became hydrophilic and negatively charged, this process altered the surface properties of microspheres, which in turn could positively affect the rate of autocatalysis of CEs. This positive feedback mediated by the properties of a larger system is the essential feature of the coding relation. Potential mechanisms of the positive feedback may include providing binding sites for new CEs and/or selective absorption of energy-rich chemicals or precursors of CEs. Simple autocatalysis is abundant in non-living nature, but it does not represent a coding relation because it has no arbitrariness or choice. Thus, besides autocatalysis CEs have to encode some function of a larger system (an organism) which would increase the success in survival and reproduction. A similar mechanism of a positive feedback through the alteration of the environment was previously proposed by Shenhav and Lancet [26]. Autocatalytic synthesis of CEs together with rare accidental collision and breakage of microspheres can lead to the propagation of the encoded properties through the entire population of microspheres. It is unlikely that any of presently known coenzymes was the CE in the first living systems. We also do not know the method of their anchoring to the oil microsphere. Possible mechanisms of binding may include positively charged groups (e.g., amino groups) attracted to negatively charged fatty acids on the surface of a microsphere (fatty acids may be products of CLM activity as described below). Alternatively, CLMs can be anchored via covalent bonds.

Oxidation of hydrocarbon terminals can be viewed as the first evolutionary step towards the emergence of bilayer lipid membranes. To make a lipid-like molecule, hydrocarbons need to be oxidized and then linked via glycerol-like molecule. If hydrocarbons were already oxidized due to the first code relation, then they became ready for the next step of making lipids, which helped to stabilize the surface of microspheres. Although glycerol can be produced abiotically, it is relatively unstable; thus, it is not clear if it can be accumulated in sufficient quantities to support lipid synthesis. Alternatively, glycerol can be produced on the surface of proto-cells by oxidation of hydrocarbons. This scenario is supported to some extent by the existence of hydrocarbon-eating bacteria [27].

It was suggested that simple autocatalytic networks can get established on mineral surface [10] or in porous walls of hydrothermal vents [3]. According to Wächtershäuser, fatty acids and lipids are synthesized as by-products of surface metabolism, which is supported by carbon fixation [10]. Although these scenarios are theoretically possible, they have several problems. First, selective accumulation of negatively charged organic molecules on positively charged mineral surface is not similar to natural selection and does not drive the evolution of metabolic networks, as claimed by Wächtershäuser [10]. Selective accumulation belongs to the non-living world and is similar to crystallization, whereas life starts with a coding relation. Thus, evolution requires an autocatalytic CE, which can modify the properties of the mineral surface so that it becomes more favorable for the autocatalysis. However, Wächtershäuser did not describe CEs, which can propagate on the mineral surface. Second, it is easier to produce fatty acids by oxidation of hydrocarbons than by carbon fixation, which requires a multi-component autocatalytic cycle similar to the reverse citric acid cycle [5]. It is unlikely that catalysts of all steps of such a cycle would appear in close proximity to each other to initiate the autocatalysis. Third, CEs have more freedom to move on the liquid surface of oil microspheres than on the solid surface of minerals. This mobility increases the rate of autocatalytic synthesis and makes the network more viable. Fourth, metabolic networks on mineral surface or in pores cannot easily invade distal niches separated by a gap or wall. On one hand, the reassembly of the carbon fixation cycle in a new location from multiple diffusing components is unlikely because the diffusion rates should be low (otherwise, the autocatalytic network is not viable). On the other hand, dispersal of the whole network on the surface of mineral particles is limited because particles are heavy and sink. In contrast, dispersal of autocatalytic networks on oil microspheres is not restricted by these factors.

One of the premises of the porous wall model is that inorganic crystals in the walls can catalyze some reactions within the network. However, oil microspheres also can carry metal ions or inorganic particles on their surface (e.g., via negatively charged carboxyl groups in fatty acids); thus, both hypotheses are equal in this respect. But inorganic particles are not likely to play the role of CEs, as it was proposed in the “genetic takeover” hypothesis [28]. Although functional information can be transferred to a different physical carrier, such transfer is possible only at a higher level of biological organization. According to the RNA world scenario, information transfer from nucleic acids to proteins started long after the origin of life, when organisms became sufficiently complex. Thus, the hypothesis of information transfer from crystals to organic molecules at early stages of life evolution (i.e., genetic takeover) is not convincing.

### 2.3. Combinatorial Coding

Hereditary system which is based on transferring of multiple types of simple (i.e., non-polymeric) CEs between parent and offspring will be called “combinatorial coding” because CEs are not connected, and hence, are transferred to offspring organisms in different combinations. Despite of random transfer of CEs to offspring organisms, combinatorial coding can be stable because (1) CEs are present in multiple copies and therefore each offspring organism has a high probability to get the full set of CEs, and (2) natural selection preserves preferentially organisms with a full set of CEs. The efficiency of the later mechanism was shown in a “stochastic corrector model” [29].

New types of CEs can be added either by acquisition of entirely new code relations, or by modification of existing CEs and corresponding code relations. It is conceivable that oil microspheres with a single type of CEs can accidentally acquire another type of CE, which encodes an additional function of the system. However, this should be viewed as a rare event because very few molecules can combine autocatalysis with additional functions within a larger system. Additional CEs have to encode novel functions to persist within primordial systems. For example, they may enhance the ability to capture energy or facilitate attachment to some substrate with beneficial consequences. The functional network of these microspheres may grow by adding new CEs and corresponding new functions. Systems with combinatorial coding have an increased evolutionary potential compared to systems with a single CE because different combinations of CEs may easily generate novel effects.

The next step towards increased evolutionary potential was transformation of old CEs into novel ones. Obviously, not every modification makes a new CE because it is necessary that the new molecule remains autocatalytic and encodes a new function. However, there may be a common core in chemical reactions, which support both the autocatalysis and the novel function. For example, it may happen that accidental methylation of the parental CE at a certain position makes a new catalyst capable of methylation. Then, this modified molecule becomes a new CE because it is capable of autocatalysis via methylation of the parental CE, and also it encodes methylation of other molecules, which is a new function. Derived CE may initially depend on the presence of the parental CE and this dependency sets the limits of recombination of CEs. However, eventually the new CE may become independent from the parental one if it acquires an alternative way of autocatalysis. Transformation can substantially increase the variability of CEs and increase the number of supported functions.

Combinatorial coding can eventually lead to the emergence of synthetic polymers. For example, if a new CE, C, can catalyze the polymerization of another CE, A, then together they encode long polymers AAAAA..., which can cover the surface of the microsphere and substantially modify its physical properties. If C can catalyze polymerization of multiple monomers (e.g., A and B) then repetitive (ABABABAB) or random (ABBAABABAAABB) sequences can be produced. These kinds of polymers may show more advanced properties including 3-dimensional folding and secondary structures. Primordial cells with polymerized CEs can substantially increase their functional repertoire, however they still have a limited hereditary potential [30] because of their inability to copy polymers with arbitrarily defined sequence. Thus, all encoded polymers at this evolutionary step were either simple repeats or random sequences. This limitation was removed with the emergence of templatebased replication, which is discussed below.

### 2.4. Origin of Universal Coding

Initial steps of primordial evolution were extremely slow and inefficient because self-production of each CE had to be developed from scratch. There was no universal rule for producing new CEs. Some improvement was achieved by transformation of old CEs into novel ones via modification of functional groups or via polymerization. In this case, at least some steps of self-production of transformed CEs could be adopted from parental CEs. However, there was still no streamlined procedure for making new CEs.

Template-based (or digital) replication is a special case of autocatalysis of CEs, where each CE is a linear sequence made of a few kinds of monomers, and copying is done sequentially via pre-defined actions applied to each monomer [29]. In theory, it may be possible to generalize the notion of template-based replication to non-linear polymers (e.g., two-dimensional sheets or branching trees), but for simplicity I will consider only linear polymers. Digital replication makes the coding system universal because the replication algorithm works for any sequence. Thus, there is no need to invent recipes for copying new or modified coding molecules. However, the notion of universal coding should be interpreted with caution because true universal properties exist only in mathematics. In the real world, there are always some limitations even within a “universal” coding system. For example, molecular machinery which is sufficient for replicating short DNA fragments (200–1000 bp) may not work for long sequences (e.g., >1 Mb). Mammalian chromosomes have extremely long DNA sequences (e.g., 200 Mb) which cannot be preserved and replicated *in-vitro*; however, mammalian cells can perform this task. Replication and elongation of telomeres also requires additional mechanisms that are not equivalent to simple template-based copying.

The starting point for the origin of template-based replication is the existence of polymeric CEs with either random or repetitive sequence. Polymers may initially stick to each other to perform some other functions (e.g., to increase stability and facilitate polymerization). Then, the shorter strand of the paired sequence can be elongated by adding monomers that weakly match to the overhanging longer strand. This step can be described as non-identical replication [31]. Then natural selection supported the increase of specificity of replication because it helped to produce better copies of existing polymers. Template-based replication probably started with copying short sequences with regular repeats, but eventually progressed into complex repeats and entirely aperiodic sequences. Invention of digital replication was the turning point in the origin of life which supported unlimited hereditary potential [30] and caused a rapid increase in the abundance and complexity of CEs. First replicating polymers were probably similar to nucleic acids; however, the sugar-phosphate backbone does not support anchoring of nucleic acids to the surface of oil microsphere. In contrast, peptide nucleic acids (PNAs) with a pseudopeptide backbone are able to absorb at the lipid-water surface [32]. Nelson *et al*. consider PNAs as possible evolutionary predecessors of nucleic acids [33].

**Figure 2.**
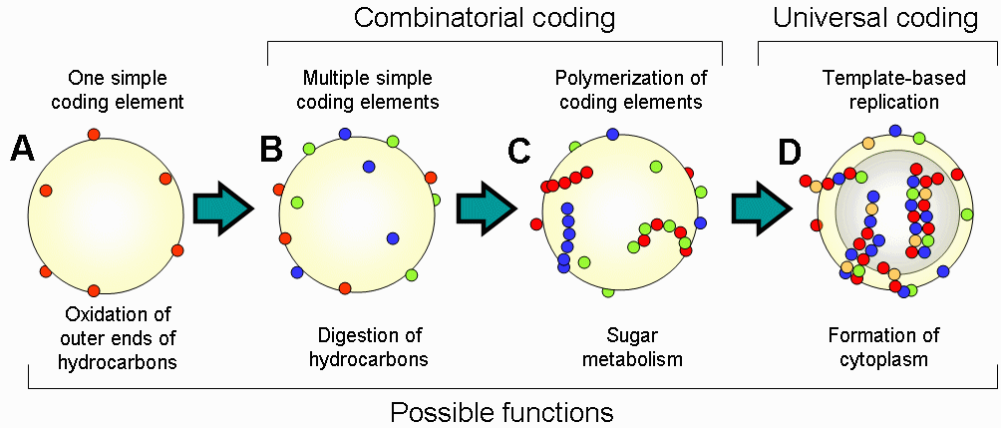
Early steps of biological evolution: (A) single coding elements; (B) multiple coding elements (combinatorial coding); (C) polymerization of coding elements (repetitive or random); and (D) template-based replication.

Major steps in the transition from the “coenzyme world” to the “RNA world” are summarized in Figure 2. Life started from the emergence of single CEs on the surface of oil microspheres; then additional CEs appeared either by capturing new autocatalytic molecules or by transformation of already existing CEs. This step of combinatorial coding was followed by polymerization of CEs and emergence of template-based replication. Complexity of the structure and function of the cell increased in parallel with the evolution of CEs. An important step of this evolution was the transition from the metabolism on the outer surface of oil microsphere to the internal metabolism. Oil microspheres with amphiphilic outer layer (micelles) can spontaneously form liposome-like structures with internal water cavities. These cavities are beneficial because they increase the ratio of outer surface used for metabolism to the amount of hydrocarbons, which is a limiting resource. Thus, any encoded changes that helped to stabilize the liposome structure were favored by selection. This evolution may have resulted in the emergence of the internal amphiphilic layer of lipid-like molecules together with ion transfer mechanisms to control the osmosis. Functional molecules started migrating from the outer surface to the inner surface, including CEs. Because the inner space was enclosed, cells started accumulating free-floating resources inside the cell, which eventually resulted in the formation of a cytoplasm. The transition of metabolism from the cell surface to the cytoplasm marks an important step towards contemporary cell architecture.

## 3. Discussion

### 3.1. Coding before Template-Based Replication

In this paper I propose a model for the origin and early evolution of living systems, in which hereditary functions were performed by simple coenzyme-like molecules (CLMs). The major difference of the proposed scenario of the origin of life from most other theories is that it does not require synthetic polymers as hereditary molecules. Previous models of life’s origin attempted to explain how complex synthetic polymers can originate from simple monomers [4,34]. Although this problem is important for explaining the increase of complexity of living systems, it is not related to the origin of life. Synthetic polymers can emerge only after accumulation of corresponding monomers. Some monomers like sugars can be synthesized in abiotic conditions; however, they are unlikely to accumulate in quantities sufficient to support viable autocatalytic networks because of low rates of their synthesis and relatively high degradation rates. Although aminoacids are more stable than sugars and are extracted from meteorites, it is unrealistic to expect that many species of aminoacids be combined in high concentrations necessary for efficient peptide synthesis. Thus, we have to admit that first living organisms did not possess any synthetic polymers. Instead, they used much simpler chemistry to encode and perform their functions. Aminoacids and nucleotides emerged and accumulated as a result of this pre-polymeric step in biological evolution, and this accumulation was a necessary condition for the later emergence of nucleic acids and proteins. Very few components of contemporary cells are remnants of pre-polymeric primordial organisms, and CLMs appear most likely candidates for performing hereditary function in those organisms.

A similar approach was recently used by Copley *et al*., who suggested a scenario of the origin of life that included phases of “monomer world”, “multimer world”, “micro-RNA world”, “mini-RNA world”, and finally “macromolecular RNA world” [3]. However, authors did not elaborate the mechanism of heredity at earlier stages (monomer and multimer worlds), and did not suggest candidate molecules that can perform hereditary functions. Because they did not consider coding relations as interactions between objects from two hierarchical levels (organisms and CEs), the role of organisms and selection was not fully acknowledged. Besides autocatalysis, CEs have to encode some useful functions of proto-organisms. In the proposed scenario, CLMs can modify surface properties of oil microspheres, which play the role of proto-organisms. Potential benefits of these changes include providing anchoring sites for new CLMs and capturing more resources (energy and precursors) for the autocatalytic network.

### 3.2. Timing of Early Evolution of Life

Functional complexity of organisms measured by the length of the non-redundant functional portion of the genome tends to increase exponentially with time, growing ca. 7.8 fold per one billion years [35]. The exponential increase in complexity can be explained by several positive feedback mechanisms, which include gene cooperation, gene duplication, and creation of new functional niches for emerging genes. This means that the evolutionary potential of living organisms increased progressively through the entire history of life. The proposed scenario of the origin of life is fully consistent with this idea. All three mechanisms of positive feedback are likely to be present in the “coenzyme world” before the origin of nucleic acids. CEs can cooperate via cross-catalysis, new CEs can be produced as modifications of already existing CEs, and the expansion of the autocatalytic network together with compartmentalization of proto-organisms creates new functional niches for emerging CEs. The greater the variability of CEs is the higher are the chances of emergence of another autocatalytic modification of some CE. Thus, we can expect that early evolution of life followed an exponential trend similar to the one observed in later evolution.

According to the exponential model, the early evolution of life was extremely slow. In particular, the “coenzyme world” stage of life may require >1 billion years of evolution because the emergence of a successful new CE without a universal coding mechanism was a rare event. First bacteria with expected genome size of ca. 100 Kb appeared on earth as early as 3.5 billion years ago [36], which leaves no time for the “coenzyme world”. Using the regression of log genome complexity versus time, the origin of life can be projected around 10 billions years ago, which implies interstellar transfer of primitive organisms (e.g., bacterial spores) within ice bodies [35]. There is no consensus on the possibility of interstellar transfer of bacterial spores, but this hypothesis cannot be rejected based on known facts [37]. If this model of biological evolution is correct, then the transition from “coenzyme world” to the “RNA world” occurred before first living organisms appeared on Earth.

### 3.3. Can We Re-Create the Origin of Life?

Experimental validation is often perceived as the only reliable evidence in favor of specific hypotheses about the origin of life [6]. Unfortunately, the proposed model of “coenzyme world” is not ready for experimental testing because the chemical nature of first CEs remains unknown. Recreating the origin of life would require a task of finding candidate molecules for the role of CEs. A possible approach is to add various organic molecules to a highly diluted oil emulsion and expect that some encoded functions will emerge spontaneously. However, it is unlikely that this approach will lead to a fast success because (1) the number of possible molecules is too large, and it may be not feasible to test all of them; (2) the mixture may appear unstable because of reactions between components; and (3) first proto-organisms may also include some ions and/or solid inorganic particles captured on the surface of oil microspheres. Testing of all possible components would substantially increase the number of experiments. Nevertheless, even if we show that first CEs can emerge spontaneously, this experiment would provide no evidence that evolution can go further. Thus, skeptics may still claim that this system is not alive and have no evolutionary potential. Experiments related to the origin of life are always engineered in a way to produce the desired effect, thus it is difficult to interpret the result as natural. However, the origin of life was a very long process [38] that possibly took billions of years, and if we do not attempt to accelerate the process, then the experiment will not be finished within any reasonable time. Thus, the only possible experimental strategy is to test one step at a time by making most favorable conditions to accelerate the process. However, re-creation of the whole process of the origin of life is definitely not feasible.

### 3.4. Coding Relation and Semiotics

Coding relation appears very similar to the triadic sign relation as defined by Peirce who wrote: “I define a sign as anything which is so determined by something else, called its Object, and so determines an effect upon a person, which effect I call its Interpretant, that the latter is thereby mediately determined by the former” (CP SS 80–81, 1908) [39]. CE can be viewed as a sign which encodes its function (=interpretant) and is determined by the organism (=object) because the autocatalysis of CEs is supported by the organism. However, not all signs are CEs. The notion of a sign is too generic and includes relations between non-living objects (e.g., a crater on the moon is a sign of a comet hit). In contrast, CEs are specific for living organisms; they have to be autocatalytic within the organism and have to encode a useful function for the organism. To encode a function, CEs need to persist for a relatively long period. Transient signs, used by organisms to cope with changing conditions (e.g., activated receptors, signal transduction pathways), are not CEs. However, the ability of organisms to generate these transient signs is pre-determined by some CEs. For example, activation of a photoreceptor by light is a transient process; however, the protein of the receptor is encoded by a gene, which is a CE. A CE can be viewed as a “mother of signs” because it can generate transient sign relations that are necessary for implementation of the encoded function.

The structure of the coding relation is consistent with previously formulated conditions of coding. Conrad viewed the origin of life as a bootstrapping process between autocatalysis and heterocatalysis [40], which are both required for CEs. However, his model does not include an organism or other kind of compartments; hence, the mechanism of evolution is not clear. Biological code can be copied and interpreted, a principle which became known as code duality [13,41]. Our model of coding relation is consistent with this principle: copying corresponds to the autocatalysis of CE (if the meaning of “copying” is generalized to include non-template mechanisms), and interpretation corresponds to the relation between CE and its function. Terms “copying” and “interpretation” imply that CEs are passive in these processes, which is not always correct. In primordial living systems, CEs were active in performing both functions. However, in later stages of biological evolution, cells developed sophisticated molecular machines to automate these processes, and the role of CEs became more passive.

## 4. Conclusions

Coenzyme-like molecules (CLMs) are most simple candidates to perform hereditary functions before the emergence of nucleic acids. By encoding surface properties of oil microspheres, to which they were anchored, they could have enhanced their autocatalysis and propagation to other microspheres. Transition from coenzyme world to RNA world is seen via diversification of CLMs, their polymerization on the surface of microspheres and subsequent development of template-based synthesis. Thus, heredity and coding emerged long before the origin of nucleic acids.
